# 

**Published:** 1845-07

**Authors:** 


### BRITISH AND FOREIGN MEDICAL REVIEW

OR

### QUARTERLY JOURNAL

OF

### PRACTICAL MEDICINE AND SURGERY

LIBRARY OF THE

Orleans Parish Medical Society

EDITED BY

JOHN FORBES M.D. F.R.S. F.G.S.

VOL. XX

JULY—OCTOBER 1845

LONDON
JOHN CHURCHILL PRINCES STREET SOHO
MDCCCXLV.

C. AND J. ADLARD, PRINTERS, BARTHOLOMEW CLOSE.

### BOOKS RECEIVED FOR REVIEW.

1. A Treatise on the Diseases and Special Hygiene of Females. By Colombat de l'Isère. Translated from the French, with additions, by C. D. Meigs, M.D. &c.—Philadelphia, 1845. 8vo, pp. 720.

2. Transactions of the Provincial Medical and Surgical Association. Vol. XIII. 8vo. pp. 418.

3. Chemistry, Meteorology, and the Function of Digestion, considered with reference to Natural Theology. (Bridgewater Treatise.) By W. Prout, M.D. F.R.S. Third Edition.—London, 1845. 8vo. pp. 516. 15s.

4. A System of Surgery. By J. M. Chelius. Translated from the German, by J. F. South. Parts I, II, III. 3s. each.

5. Contributions to the Medical History and Treatment of sexual Diseases. By J. H. Robertson, M.D.—Glasgow, 1845. 8vo, pp. 80.

6. Sixth Annual Report of the Registrar General of Births, Deaths, and Marriages in England.—London, 1845. 8vo, pp. 666.

7. Outlines of Chemistry for the use of Students. By W. Gregory, M.D.—London, 1845. 8vo, pp. 588. 12s.

8. Repertorium der K. K. Oesterreichischen Medicinal-Verordnungen. Von T. Jurié, M.D.—Wien, 1843. 8vo, pp. 250.

9. Observations on the Education and Examination for Degrees in Medicine, as affected by the New Medical Bill. By Richard Quain, F.R.S. —London, 1845. 8vo, pp. 60.

10. Handbuch der Heilmittellehre. Von Dr. Fr. Oesterlen.—Tubingen, 1845. 8vo, pp. 1052.

11. The Physiology of Digestion considered with relation to the principles of Dietetics. By Andrew Combe, M.D. Fifth Edition, revised and enlarged.—Edinburgh, 1845. 8vo, pp. 154. 2s. 6d.

12. Glanders and Farcy in the Horse. By W. Percivall, Surgeon.—London, 1845. 8vo, pp. 352.

13. On the present state of Therapeutical Inquiry. By James Arnott, M.D.—Brighton, 1845. 12mo, pp. 58.

14. Objections to Animal Magnetism, with remarks on Mr. Hall's Lectures.—Reading, 1845. 8vo, pp. 24.

15. The Power of the Soul over the Body, considered in relation to health and morals. By G. Moore, M.D.—London, 1845. 8vo, pp. 306. 7s. 6d.

16. A Practical Treatise on the Special Diseases of the Skin. By C. M. Gibert. Second Edition. Translated from the French by E. Sheppard, Surgeon.—London, 1845. 8vo, pp. 362. 7s. 6d.

17. The Principles and Practice of Dental Surgery. By C. A. Harris, M.D. Professor of Dental Pathology in Baltimore. Second Edition. —Philadelphia, 1845. 8vo, pp. 600.

18. Observations upon the Employment of Compression in Aneurism. By O'B. Bellingham, M.D.—Dublin, 1845. 8vo, pp. 14.

19. Vestiges of the Natural History of Creation. Fourth Edition.—London, 1845. 8vo, pp. 408. 7s. 6d.

20. Memoir of the Life of a Country Surgeon. —London, 1845. 8vo, pp. 22.

21. The London Medical Directory, 1845.—London, 1845. 8vo, pp. 180. 5s. 6d.

22. A Guide to the Use of the Buxton Waters. By W. H. Robertson, M.D.—London, 1845. 12mo, pp. 32.

23. The Gymnastical Orthopœdical Institution in Dessau, its management and efficaciousness. By Dr. J. A. L. Werner.—Dessau, 1845. 8vo, pp. 48.

24. Observations on the Mechanism and Diagnostic Value of the Friction Vibrations in Peritonitis. By R. Spitral, M D. (From the Edinb. Monthly Journal.) 1845. 5vo, pp. 20.

25. Observations on the Nature and Treatment of the more important Diseases of the Nervous System. By a Physician. (Dr. Blackmore.)—Bath, 1845. 12mo, pp. 90.

26. Prize Clinical Reports of Surgical Cases treated at the Queen's Hospital, Birmingham. By John Moore.—London, 1845. 8vo, pp. 122. 2s. 6d.

27. Practical Notes on Insanity. By J. B. Steward, M.D.—London, 1845. 8vo, pp. 65.

28. Remarks on Physicians, Surgeons, Druggists, and Quacks. By Surgeon Snipe.—Halifax, 1845. 8vo, pp. 66.

29. Mental Maladies. A Treatise on Insanity. By E. Esquirol. Translated from the French, with additions.—Philadelphia, 1845. 8vo, pp. 496.

30. Hindustani Version of the London Pharmacopœia, ed. 1836. By G. G. Spilsbury, Surgeon, and SamachurnDutt, sub-assistant surgeon, 1841. Done into the Persian, with an Appendix. By F. J. Mouat, M.D.—Calcutta, 1845. 8vo.

31. Guy's Hospital Reports, April, 1845. 6s.

32. Practical Remarks on some exhausting Diseases, particularly those incident to Women. By Sir James Eyre, M.D.—London, 1845. 8vo, pp. 75. 4s.

33. Manuel Topographique et Médicale de l'Etranger aux Eaux d'Aix-en-Savoie. Par C. Despine (fils), M.D.—Anneci, 1843. 8vo, pp. 234.

34. Seven Lectures on Somnambulism. Translated from the German of Dr. Wrenholt, with Notes, &c. by J. Colquhoun, Esq.—Edinburgh, 1845. 12mo, pp. 220.

35. The Seeress of Prevorst. Translated from the German by Mrs. Cooke. 1845. 8vo.

36. Systematische Darstellung des Medicinal-Dienstes des Oesterreichischen Kaiserstaates. Von Dr. J. Müller.—Wien, 1844.

37. Remarks on Clause 38 of Sir JamesGraham's Medical Bill.—London, 1845.

38. A Case of Cæsarean Operation, with remarks on Puerperal Metritis. By J. M. Coley, M.D.—London, 1845. 8vo, pp. 30.

